# Involvement un-enabled? An ethnographic study of the challenges and potentials of involving relatives in the acute ambulatory clinical pathway

**DOI:** 10.1186/s12913-020-05923-x

**Published:** 2020-11-26

**Authors:** Susanne Nissen Sagoo, Regine Grytnes

**Affiliations:** Department. of Occupational Medicine, University Research Clinic, Regional Hospital West Jutland, Gl. Landevej 61, Herning, Denmark

**Keywords:** Relatives’ involvement, Emergency care, Clinical pathway, Ethnography

## Abstract

**Background:**

Involving a patient’s relatives is a complex endeavour, especially in emergency departments (EDs). Generally, relatives are recognized as vital partners in health care, but in-depth knowledge on how these family involvement processes take place in the everyday practices of EDs is sparse. The aim of this study is to explore the practice of involving relatives in the acute ambulatory clinical pathway in the ED, as seen from the perspectives of patients and relatives.

**Methods:**

The study was conducted as ethnographic fieldwork in an ED at a Danish Regional Hospital. Two months of participant-observation were carried out focusing on 43 patients. Of these, 18 patients and/or relatives were selected for telephone interviews after 1 week, and of these 11 were selected for in-depth interviews 3 weeks later.

**Results:**

Unpredictability is a basic condition of any ED. For the patients and relatives, who are unfamiliar with the routines in the ED, unpredictability translates to a sense of temporal and existential unpredictability, reinforced by a sense of not knowing when the examinations will be completed or if/when they will be sent home. Relatives’ involvement in the ED is affected by this sense of unpredictability and by the existing relations between patients and their relatives prior to entering the ED. The stay in the ED is only one ‘stop’ in the complete acute ambulatory clinical pathway but relatives’ involvement also concerns the time before and after the stay in the ED. Practices of involving relatives leave (some) relatives invisible in the clinical pathway. As a consequence, they are often not addressed, which *un-enables* their involvement.

**Conclusion:**

Involvement of relatives presupposes recognizing the relatives as participants if they are to be involved in the patient’s clinical pathway in the ED. As a start, it is advisable that the medical staff ask the patients on arrival who has accompanied them in the ED, and if and in what way they want their companions involved in the ED. There is a need for a more integrated and contextualized understanding of relatives’ involvement, as it takes place along an extended acute ambulatory clinical pathway.

**Supplementary Information:**

The online version contains supplementary material available at 10.1186/s12913-020-05923-x.

## Background

### Knowing the patient and their relatives

Well into the fieldwork in the Emergency Department (ED) on which this study is based, one of the nurses introduced a patient, named Kate (a pseudonym) whom she thought should be included in the study. Unlike the other patients in our study, we had received some information about Kate before the first author met her. The nurse had worked with Kate’s daughter and the community health services in planning Kate’s discharge from a previous hospital stay. The nurse knew that Kate was fragile, and she advised me to bring a small fruit drink to offer Kate, as she could ‘easily get a little upset and a bit angry, and if you bring her a drink it might help to calm her’ (all translations from the Danish by the authors).

This fieldwork experience of being guided by the ED nurse in order to approach Kate in the most gentle way became a mirror with which we viewed important aspects of studying the practice of involving relatives in the ED. What the nurse did was to introduce Kate as a person with whom she had already established a relationship; she could therefore anticipate how Kate would react, for example, when a new person (I, the researcher) approached her. This anecdote illustrates the privileged information sometimes obtained in fieldwork as well as the difficulties the medical staff encounter when patients and their relatives are received in the ED and when information about ‘their history’ is sparse or difficult to obtain in the course of the acute clinical pathway. Advice such as ‘bring a fruit drink’ is usually not written in the patient’s medical chart, but it is occasionally available as tacit knowledge [1, 2] or from the patient’s relatives. *Knowing* is, in this case, more than getting information on a patient’s disease or medication, knowing also concerns information about the patient as a person, information that might prove relevant in terms of understanding the relatives’ involvement.

Literature on the importance of involving patients’ relatives in clinical pathways [3–5] is gaining increased attention, both internationally as well as in Denmark. In a health care context, it is the patients themselves who identify their relatives, e.g. family (e.g. spouse, parent, sibling), friends, neighbours, or other relations [6]. From an institutional point of view, involving the relatives entails cooperating with them in the patient’s ‘clinical pathway’, which is defined as all the ‘activities, contacts or events that patients go through in connection with the treatment of a certain health condition from the first to the last contact with the health care system’ [7]. Relatives are recognized as vital partners in improving the quality and safety of healthcare and in creating coherent clinical pathways [3–5, 8–10]. Research has pointed out that benefits of involving relatives in healthcare include the improvement of patients’ knowledge and understanding of their own health and the treatment they receive, ensuring the patient’s adherence to treatment, patient safety, patient and professional satisfaction, practice efficiency, as well as the reduction of the costs of health care [6]. In the Danish healthcare system, protection of the patient’s legal rights is enshrined in the Danish health legislation, and accordingly, healthcare personnel are obliged to maintain confidentiality, also toward the patient’s relatives. Therefore, in a legal sense, patients must give their consent if health personnel are to provide their relatives with confidential information about them [11]. Even if they have given consent to the involvement of their relatives, the involvement of relatives can create conflict, as patient and relatives may have different perceptions of the patient’s situation and of the kinds of treatment they think should be performed [12, 13].

Also, there are many different types and levels of relative involvement [14], which means that involvement is not simply a question of being involved or not. Relatives may be involved in contributing important information about the patient’s personal or medical situation, helping the patient with practical, social, and emotional support, as well as acting as coordinators, advocates, and family caregivers for the patient in his or her clinical pathway [15–17]. Despite the possibility of differences of perspectives, there is no dispute as to the importance and benefits of involving relatives in the clinical pathway.

Nevertheless, involving relatives in the ED can be a challenging task. The time pressure in the ED is often more intense than in other hospital wards [18], and the medical staff must ensure that patients are diagnosed, treated, and discharged or admitted to another ward as quickly as possible [19]. This has been referred to as the ‘flow culture’ [20]. Consequently, the medical staff in the ED ward have little time to build relations, obtain necessary information, and assess the patient’s situation [18]. Studies have also shown that involving relatives is challenging because relatives might not be present during the patient’s visit to the ED [17, 21]. Some patients do not want their relatives to become involved [21], some patients arrive alone [16, 17, 19, 21] and some relatives are only temporarily present during the patient’s visit to the ED [17]. At times, relatives who are in fact present and willing to be involved find that the nursing staff excludes them from being involved in the patient’s clinical pathway [22]. Relatives may feel that it is their own responsibility to take the initiative if they want the nurses to involve them in the patient’s treatment process [23]. Therefore, although it is important to involve relatives in patients’ clinical pathways in order to enhance quality and safety, this task is far from easy. The time pressure and the flow culture add to the complexity because the staff may understandably have difficulty finding the time to involve the patients’ relatives. This raises the question of how relatives can get involved and what kind of involvement of relatives is needed in the environment of the acute ambulatory clinical pathway in order to gain the potential benefits of relatives’ involvement.

In-depth knowledge on how relatives’ involvement proceeds in the everyday practices of the ED is sparse. The aim of this study, therefore, was to explore the practice of involving relatives in the acute ambulatory clinical pathway in the ED as seen from the perspectives of patients and their relatives. Our goal was to understand why involving relatives is so difficult in the clinical pathway and to investigate whether the degree to which the relatives’ involvement practices are premised on and related to other practices in the acute clinical pathway.

## Theoretical framework

To examine the practice of involving patient’s relatives, we drew on practice theories [24–26]. From this perspective, practice is understood to be a set of individual and social doings and sayings organized by means of different constituent elements such as procedures, statements, understandings, knowledge, engagement, the body, and objects, all of which work in mutual relation with each other [25]*.* By focusing on the participants ‘doings and sayings’, we zero in on how individuals are given the opportunities to participate in the social practices of involvement and to analyse the integration of different practices that constitute the specific practice of involving relatives.

At the organizational level, a hospital can be seen as a place where ‘multiple practices are carried out at the same time’ ([26] , p. 2). A hospital or hospital department is not an isolated entity. Practices inside the hospital build upon other related practices, e.g. in Denmark a practice of referring patients from general practitioners (GPs) to the ED, or from the ED to other hospital departments. On that basis, our focus is to investigate how individuals ‘become participants’ and how they ‘subsequently perpetuate and transform practices through their actions’ ([27] , p. 6). All practices are adaptable, and the individual performance is situated in the context of a particular practice; a practice ‘takes place and is intelligible only as part of an ongoing practice’ ([27] , p. 14)*.* Thus, an analysis from a practice theoretical perspective does not focus on individual actions as such, but rather on what has been referred to as the *scenes of action* ([26] , p. 7) in which the individuals could act.

The concept of *enablement* [28] has proven especially useful in opening up the analysis of involving patients’ relatives. Enablement ‘indicates that people only become carriers of specific abilities through participation in practices’ (ibid., p. 9). It also suggests that ‘the status of a participant is dependent upon mutual recognition’ (ibid.) from other participants*.* Participants (patients, relatives, physicians, nurses, and ambulance personnel) take part in a practice from their different social positions and bring with them different perspectives. The concept of enablement highlights the importance of questioning exactly how patients, relatives, and the professional actors become enabled to support each other through their mutual recognition of each other ([28] , p. 12). In relation to this study, to highlight how this mutual recognition does not always take place, we use the word *un-enablement*, to refer to practices by which patients, relatives, and the professional actors in the clinical pathway fail to recognize each other and thereby do not help, inform, or make each other capable of taking part in the clinical pathway.

Understanding the process of involving patients’ relatives as a practice is thus a ‘way of looking at how patients, relatives and health professionals initiate themselves and make themselves into participants by equipping one another with the collaboration of things and artefacts, with situational possibilities of action and, at the same time, delimiting them’ ([28] , p. 12). The development of competent participation in this setting is therefore understood as a process in which specific knowledge, identity, and social membership are thus embedding ‘the practice of involving relatives’ within power relations and conflict ([28] , p. 9). In order to be able to study the development of involvement practices in this manner, ethnographic fieldwork in the ED was conducted, as described below.

## Methods

This study was designed as a qualitative in-depth single case study in an ED at a public Danish Regional Hospital [29]. It was conducted as ethnographic fieldwork (by the first author) and comprised of participant-observations at the department and interviews with patients and their relatives [30]. The ED receives many different kinds of patients with a broad range of medical conditions of different severities. We thus applied information-oriented selection methodology, selecting participants using purposeful sampling aiming for maximum variations in the information content [29].

### The field

Access to the ED was obtained through a charge nurse, who consulted the ED management about the project to which they agreed. The ED where we carried out our ethnographic study is composed of an emergency admission and an emergency bed ward. The hospital is a part of the tax funded public health care system and has a catchment area of approximately 300,000 persons and covers six different municipalities. Around 29,000 patients come through the ED annually, of whom approximately 33% require in-patient examination and treatment.

Before the start-up of the participant-observation, a newsletter was posted to all members of staff in the ED describing the project and the researcher. In addition, during the first 3 days, the first author introduced the project and herself at morning meetings in the ED, but individual consent from the staff was not obtained. The first author wore non-medical attire with a visible name badge which indicated her status as ‘Researcher’. Most of the nurses and physicians knew the first author from prior research projects in the ED.

The study focused primarily on adult patients (18 years and older) who were not critically ill (e.g. cardiac arrest) or who had minor injuries (e.g. fractured finger). Patients were eligible for the study regardless of whether their relatives were present at the ED or not. At the beginning of the fieldwork, we relied on a physician’s^1^ assessment of whether a patient was eligible for the study. To be able to investigate a range of situations and problems, patients were selected to ensure an equal gender distribution and a range of ages, diagnoses, and medical conditions.

#### Participant-observation

Participant-observation was conducted during 1 month in the spring and 1 month in the autumn of 2017. On average, the first author spent 8–12 h each day at the department, which amounted to about 200 h in total. As the ED operates around the clock, the participant-observation was carried out on weekdays, weekends, and on public holidays, at all hours.

The relatives selected for participant-observation and eventual interviews were recruited through the patients – either during the patients’ stay in the ED, or later on, in the interview phase. If the patients were unable to give their consent due to medical conditions or personal issues, the relative decided whether the patient and they themselves would participate. Patients were recruited as soon as possible after the patient’s arrival in the ED; patients arrived at the ED by ambulance, were transported by relatives, or transported themselves. The patient, and, if present, the relatives, were informed about the purpose of the observations and asked (non-standard language) to give verbal consent to allowing the researcher to stay with them during their stay in the ED. Of those asked, no one rejected.

The participant-observation was conducted as the patients underwent medical examinations, waited in their beds or in the waiting room, small talking or helping out the patient and/or the relatives. In all, 43 patients and their relatives were followed. Further data about the patients is shown in Table 1.

**Table 1:** Participants characteristic

Patients characteristics		Reason for referral	Trans-portation	Relatives present?	Admitted to/sent home/transportation	Telephone interview	Semi-structured interview
Male	60 - 65	Skin infection	Taxi	No, wife is at work	Stayed overnight, intravenous antibiotic/ taxi	Wife	Patient and wife
Male	65 - 70	Fell on the floor , syncope	Emergency Services	No, decided that his wife should remain home	Sent home		
Male	75 – 80	Vomit, chest and stomach pains, abstinence	Emergency Services	No, did not want his son and daughter involved	Stayed overnight/ taxi	Patient	Patient and daughter, separately
Male	80 - 85	Had a fall, deep cut in his forehead	Ambulance	Wife	Stayed overnight, sent to respite care/ ambulance	Wife	Wife
Male	70 – 75	Severe pain in his legs, rheumatic disease	Ambulance	Wife	Stayed overnight, antibiotics, walked home with wife	Patient	Patient and wife
Female	65 - 70	Fell down the stairs, checked for a ruptured spleen	Son	Son and grandchild	Sent home with son		
Female	75 - 80	Had a fall, cut on the leg	Ambulance	Partly, both daughters came after 1½ hours	Sent home/ambulance	Daughter	
Male	45 – 50	Suspicion of blood clot in his leg	Patient	No, single	Sent home/patient		
Male	65 - 70	Felt unwell, pacemaker	Emergency Services	No, wife chose not to accompany the patient	Sent home/wife	Patient and wife	Wife
Female	75 - 80	Fractured her wrist	Daughter	Daughter	Sent home/daughter		
Female	15 – 20	Had a fall, suspicion of concussion	Ambulance	Friend. Parents came when the patient was ready to go home	Sent home/parents		
Female	45 - 50	Suspicion of blood clot in her leg	Patient	No, her husband had returned home from night shift	Sent home/patient	Patient	
Female	20 – 25	Suspicion of fractured spine	Ambulance	Mother	Sent home/mother	Mother	Patient and mother, separately
Female	70 – 75	Had a fall, fractured wrist	Grandchild	Grandchild	Sent home/Grandchild		
Male	30 – 35	Suspicion of appendicitis	Mother	Mother	Sent home/mother	Patient	
Male	65 – 70	Stomach pain, Cancer patient	Ambulance	Yes, wife, arrived later because of work	Admitted to cancer department		
Male	60 – 65	Had a fall, briefly unconscious	Ambulance	Partly, son-in-law, the patient sent him home after a short while	Sent home/son in law		
Male	65 - 70	Fever, cancer patient	Ambulance	Wife	Admitted to cancer department		
Female	65 – 70	Vomiting	Ambulance	No, lived alone	Admitted to dept of internal medicine		
Female	85 – 90	Atrial fibrillation	Emergency Services	Yes, partly, daughter after two hours	Sent home/daughter	Patient and daughter, separately	
Female	75 – 80	Had a fall, suspected concussion	Ambulance	Friend (patient was visiting her friend)	Sent home/another friend		
Male	35 – 40	Stress	Emergency Services	Wife	Sent home, wife		
Female	80 – 85	Suspected embolus in the lung	Daughter/ambulance	Yes daughter, partly, went home after a few hours	Admitted to department for heart diseases		
Female	75 – 80	Atrial fibrillation	Husband	Yes, husband, daughter (nurse) and sons came shortly after her arrival	Admitted to department for heart diseases	Patient and daughter, separately	Daughter
Female	65 – 70	Suicide attempt	Ambulance	Husband, son-in-law	Stayed the night /husband		
Female	75 – 80	Feeling unwell, suspicion of heart problems	Ambulance	No, her husband stayed home	Sent home/husband		
Female	25 – 30	Stomach ache	Patient, with train	No, fiancé was at work	Sent home/patient went home by train		
Male	35 – 40	Fainted because of violent stomach pains	Emergency Services	Yes, wife came after a relative had come to look after the children	Sent home/wife		Wife
Female	55 – 60	Fractured wrist	Neighbor	No, single	Sent home/taxi	Patient	
Female	75 – 80	Fall, pain from knee	Ambulance	No, patient sent son to work	Sent home/taxi	Patient	
Female	90 – 95	Severe bleeding	Ambulance	Yes, partly, daughter came later on			
Female	65 – 70	Stomach ache, terminal cancer	Ambulance	No, husband was not present	Admitted to another department		
Male	90 – 95	Felt unwell and was not able to stand on his feet	Ambulance	Yes, wife and son came later on	Admitted to the intensive department	Son	Son
Male	40 – 45	Fainted and bumped his head	Ambulance	No, father at home with the patient's children	Admitted to another department	Patient	
Male	65 – 70	Rupture of Achilles tendon	Wife	Wife	Sent home/wife		
Female	40 – 45	Dislocated shoulder	Patient	No, husband at work	Sent home/researcher and work colleagues	Patient	
Female	50 – 55	Stomach ache	Husband	Husband	Sent home/husband		
Male	85 – 90	Heart?, pain from shoulder	Ambulance	Son	Sent home/son		
Female	100 – 105	Emergency service referred her to the ED, worried about her condition	Ambulance	Son	Sent home/ambulance		
Female	75 – 80	Wound on tibia	Daughter	Daughter had accompanied the patient, but has left to go home	Stayed the night over		
Male	70 – 75	Fractured clavicle	Ambulance	No, Relatives are not capable of taken on the role of a relative	Stayed the night over, send to respite care		
Female	75 – 80	Feeling unwell, suspicion on deterioration of existing disease	Ambulance	No, daughter is unwell			

#### Interviews

Apart from the observations and informal interviews in the ED, telephone interviews were conducted with 18 patients and/or relatives. Patients from the two largest municipalities in the catchment area were chosen. In these interviews, focus centered on the patient’s and/or relative’s reflections on their stay in the ED and on the patient’s situation after returning home. Eleven patients and/or relatives were selected for additional interviews, which were conducted around three weeks after the telephone interview in the patient’s home or at the first author’s workplace. The criterion for selecting patients for the semi-structured interviews was that they should represent different cases of relatives’ involvement. As a rule, participants were selected from the group who participated in the telephone interviews, with a few exceptions. The interviews lasted on average 1½ hours, with the patient and/or relative granting written consent. The interview guide consisted of open-ended questions constructed on the basis of the pre-knowledge from the participant-observation phase and the telephone interviews as well as the scientific literature. The interview guide designed for this study is provided as Additional file 1. All data was anonymized, and all names used in this article are pseudonyms.

### Data analysis

The overall analytical approach is ‘interpretive looking’, which requires an abductive logic of reasoning characterized by an iterative and recursive process, not only across data sources in the field, but also between theoretical and field encounters as well as a focus on contextual meaning [31].

The analysis of data began at the start of the fieldwork period and was a continuous process through the entire project and not solely in the analysis phase. In anthropological fieldwork, the researcher writes up thick descriptions that are not only observations from the field but also include the researcher’s own reflections and experiences. This means that each period of participant-observation builds upon experiences/data from the previous period. This is illustrated by the development of the questions asked, as the knowledge from previous encounters with patients and relatives is used when asking questions or performing actions in the following encounters. Likewise, the same method is used in constructing the interview guide as well as conducting telephone interviews and semi-structured interviews. Data therefore consist of field notes, notes from telephone interviews, as well as recorded and transcribed semi-structured interviews.

The recorded data was read and reread to identify themes and narratives in each case and across the different cases. The transcripts were not returned to the participants but were discussed with the project’s steering group. The data was coded thematically and categorized into themes and subthemes [32], and on the basis of notes from participant-observation, telephone interviews, and semi-structured interviews, ‘ethnographic tales’ [33] were constructed. This analytic process involved reading all the material and identifying themes. On this basis, we attempted to ‘translate’ the material in an interpretive manner (ibid.). Ethnographic validity is thus created through the credibility of the text: does the text appear authentic, nuanced, and convincing, and does it relate to the context? The ethnographic tales were read and analyzed across the individual cases by both authors. For the purpose of this article, we use the understandings achieved from all the ethnographic tales of the individual patient’s acute clinical pathway, and examples of the most exemplary cases are used in the presentation of the results in order to illustrate how involving patient’s relatives in the ED acute clinical pathway is practiced and what this means for quality and patient safety.

## Results

### Part 1: unpredictability in the acute ambulatory clinical pathway

In this section, we examine the functioning of and practices in the ED as viewed primarily from the perspective of patients and their relatives; the way in which patients were received, examined, diagnosed, treated, and sent home were important in terms of determining the opportunities to practice involvement in relation to relatives in the acute clinical pathway in the ED.

Unpredictability is inherent to emergency care. It relates to the fact that it is difficult to predict when acute patients will be referred to and received in the ED, and to the clinical pathway in the ED. Even in cases where the ED has been notified about the referral of a patient, they do not know exactly when the patient will arrive or the severity of their condition. The results of the participant-observation show how this unpredictability resulted in continuous adjustments by the medical staff, which can be illustrated in the following quote from the field notes:

*From the staff’s break room, I am able to watch what is going on in the adjacent corridor. There is access to several of the treatment rooms and to the main office in the ED. All patients who arrive by ambulance pass through this way. Suddenly, I observe an increased activity. I feel the intense atmosphere. The staff is moving patients out of some of the treatment rooms. I wonder what is happening. Shortly afterwards, I get the answer when ambulances arrive and one patient after another is wheeled into the just vacated treatment rooms. A nurse tells me that there has been a traffic accident with multiple cars involved.*

This observation illustrates that it is not only the number and sequence of patients that is unpredictable, but also the variation and severity of their emergency conditions. For the staff, this unpredictability requires a certain routine regarding the admission of patients to the department. One physician explained that around 11:00 am, patients who had seen their GP in the morning started to arrive. The practice of having to see a GP before arriving at the ED^2^ therefore affected the routine practices of the physicians. During their rounds in the bed ward, when they would decide who was ready to be discharged or who needed to be transferred to other departments, the physicians had to keep in mind that new patients would be arriving around noon. This decision-making practice of ‘keeping beds free’ in order to be able to receive incoming patients is a way of meeting the challenges of the unpredictable. Free beds are a sparse resource in the ED, and the efficient and competent management of free beds enables the patient flow to operate smoothly: to receive, examine, treat, diagnose, and send patients home or to further treatment elsewhere. If this flow is not maintained, a bottleneck might develop, and the ability to keep the flow running and to control the unpredictable inflow of patients (as with the traffic accident referred to above) will be jeopardized.

Adding to this, we found that there was an inherent unpredictability for the patients related to the examinations, diagnosis, and treatment in the ED. These procedures were understood very differently by physicians and nurses compared to the patients and their relatives. This difference in understanding related especially to the timeframe of these procedures. Several of the patients and relatives were thus caught by surprise when they realized that their examinations had been completed and they were being sent home. The informal interviews with the patients and relatives revealed that this uncertainty created a sense of temporal unpredictability that made it difficult for them to navigate their involvement in the ED. For example, some of the patients told their relatives to go home because they foresaw a long waiting time, additional examinations, or monitoring of their condition, only to realize shortly afterwards, that their examinations had been completed and that they were free to leave. An example of this was Beth, an elderly woman who was referred to the ED on suspicion of a deep vein thrombosis in her leg by a doctor from the emergency medical services. Beth arrived alone in the ED, as she did not want her son to accompany her; he had to go work. Beth explained that she was also recovering from recent back surgery and therefore had walking aids at home and was about to begin rehabilitation therapy with a physiotherapist soon. When the results of the X-ray examinations showed no deep vein thrombosis, the attending ED physician told her that she should go home. However, he also discussed with Beth her rehabilitation plan and referred her back to the physiotherapist. However, Beth felt that this was insufficient and that she needed further examinations, as is evident in the following excerpt from the field notes:

*The nurse, who is seeing Beth for the first time, rushes into the room and walks up to Beth. She tells Beth that she will help her to go home. Beth, who is not at all happy about the physician’s decision to send her home, tells the nurse that she feels that she has not been sufficiently examined. She voices dissatisfaction with the physician’s decision and that, if she has to go home, she wants to go home the same way as she arrived, by ambulance. The nurse leaves the room to talk to the physician. I (first author) think that Beth had hoped to be hospitalized, because she felt that she was not ready to go home, where she lives alone. When the nurse returns, she reconfirms the physician’s decision because the examinations have shown that nothing is wrong. The nurse also tells Beth that she is not entitled to transport by ambulance if she is able to sit in a taxi. The nurse then helps Beth up and out of the bed and assists her to sit down in a chair before she leaves the room to call for a taxi. Beth tells me that she feels unsafe, but she has some neighbours whom she can call for help when she is home.*

A week later, in a telephone interview, Beth was still very angry with the physician’s decision, as it created a sense of insecurity and vulnerability during her discharge. Nevertheless, she stated that things had turned out fine at home and she had been able to manage with the help of her neighbours. The different perceptions of the practices of ‘examining, diagnosing, and treating’ are evident in Beth’s case, but it was not a routine practice to remedy this in the clinical practice. Patients, understandably unfamiliar with the practices related to examining, diagnosing, and treating arrivals in the ED, do not have the knowledge of how these, often unpredictable practices take place. This is in contrast to the staff, who rely on their daily routines and know the ED, patients have few ways to remedy this unpredictability. In addition, in Beth’s case, when the physician had treated the acute medical condition based on specific examinations (the X-rays and his physical examination) he also took time to talk to Beth about her rehabilitation plan and discussed referring her back to her physiotherapist. It was obvious that he assessed Beth’s situation as related to her back surgery and not a new condition in her leg. Seen from Beth’s perspective, however, she was concerned mostly about this new condition; thus, she left the ED feeling that she was insufficiently examined, with the added ‘insult’ of being placed in a taxi instead of an ambulance.

We also found that in some cases, the patient’s relatives acted in order to reduce the felt unpredictability of the patients and themselves. One way of reducing this unpredictability was to obtain knowledge about what would happen to the patient. This is illustrated in the case of Mary. She was an elderly woman, who was admitted to the ED suffering from hypertension and she was lying in the bed when her daughter Liza came to visit. It turned out that Liza worked as a nurse in an inpatient unit that specialised in her mother’s disease. When Liza arrived, she quickly scanned all the different monitors and went looking for the physician in charge of Mary’s examination. On returning, Liza was able to explain the status of Mary’s condition and reassure Mary, her father and siblings that everything was under control. By acting as she did, Liza set aside the physician’s usual practices of informing the patient of the examination results directly. Because Liza was recognized as a nurse, the physician informed her of the results of Mary’s examinations sooner than he normally did. Being a registered nurse enabled Liza to act in ways that not many relatives are capable of. This fieldwork experience provided a valuable perspective on different positions available to relatives and functioned as a mirror through which we became aware of some of the advantages of being a registered nurse family caregiver [34] when it comes to relative’s involvement.

Above, we have contextualized the inherent unpredictability in the acute ambulatory clinical pathway in the ED, and we have shown how the practices of unpredictability in the ED are conditioned by practices outside the ED. This suggests that unpredictability constitutes one of the core framework conditions of the functioning of the ED. Unpredictability means different things and has different manifestations for the ED staff and for the patients and their relatives. For the ED staff, the unpredictability operates in terms of how and when the acute patients arrive in the ED, and this unpredictability of intake influences how they administer resources such as beds and time. The ED staff practices are influenced by practices and events outside the department: the referring practice of GP’s, the occurrence of large accidents, and the ED staff’s ability to refer their patients to out-patient treatment. Unpredictability for the ED staff is thus translated into routines of keeping beds free so as to be able to continuously receive incoming patients and keep the patient flow running smoothly. For the patients and relatives, (mostly) being wholly unfamiliar with the routines in the ED, the unpredictability manifests itself as temporal and existential lack of knowledge: not knowing when the examinations have been completed, not knowing whether they are being discharged, or how their diagnosis (or lack of it) will affect their future.

In the following, we turn to the conditions affecting the practices of involving relatives in the ED treatment context.

### Part 2: involvement and un-enablement

Across the data, we found that the practices of involving relatives could not be isolated to the patient’s stay in the ED; rather, these practices of involvement between the patient and his or her relative(s) were part of on-going, already established relationships and practices. These already established practices play a role in how patients and relatives act within the ED setting. As such, these practices might involve experiences from prior visits to the ED or other hospital departments, and play a role in how patients and relatives act within the ED setting, such as whether to involve relatives.

Involving relatives in the patients’ clinical pathways therefore depends on how the participants have been enabled to act in their own specific pathways. In the following, the practices regarding involvement before, during, and after the patient’s stay in the ED will be exemplified through three cases: Paul’s case, in which no relatives were able to take part but where their role later becomes crucial; Debbie’s case, in which her mother was present but hardly involved; and John’s case, in which John is reluctant to involve his daughter.

#### A scene where no relative could act

The patients’ relatives were present at different times and in various times intervals during the patients’ stay in the ED. Moreover, it was not always the patient’s closest relative who accompanied them to the ED. It might also have been someone who was present at the time of the accident. Even though a patient had close relatives who might want to be involved in the patient’s clinical pathway, they might be unable to fill this role because they were at work, lived far away, or were themselves sick or disabled. This can be illustrated in the case of Paul. Paul, who was in his seventies, had a fall in his home, where he was found by personnel from the community health services that had come to assist his ill wife. The health workers called for an ambulance, and Paul arrived alone in the ED late on a Sunday night. The nurses and physicians in the ED were informed by the health workers that Paul was suffering from dementia. During the participant-observation, Paul seemed very social and told me stories from his life, but when the physician entered the room, it became readily apparent that he had difficulties remembering. An excerpt from the field notes:

*The physician walks over to Paul’s bedside. He asks Paul: ‘How did this happen?’, and Paul answers: ‘I don’t know.’ The physician then looks at me, and I have to tell him ‘I don’t know.’ He asks Paul several questions, but Paul is unable to answer any of them, including the questions about where he has hurt himself. Every time his answer is: ‘I don’t know.’ Later on, the physician returned and asked Paul about his medications, and each time Paul replied: ‘I don’t take any medication.’*

This conversation illustrates that the absence of a relative who knows Paul, his condition, and what had happened before he arrived at the ED resulted in a lack of knowledge about Paul. As a participant-observer spending time with Paul, I was able to find out about Paul’s family and situation because I spent time sitting at his bedside and listened to him when he talked about things that came to his mind. I was also able to observe how he constantly moved his right arm, and sensed that he had a disagreeable or painful sensation which he was unable to articulate. Later on, the physician returned and told Paul that his collarbone was fractured and that they were giving him a support bandage to keep him from moving his arm. Shortly after the physician had left, Paul started to move his arm again, and I went over to his bedside. When I told Paul that he had fractured his collarbone, he looked at me rather puzzled and said *‘*Really?’ I also told him that he had to keep his arm still. Paul stopped moving his arm for a short while, until he moved it again. This happened repeatedly until the nurse wrapped the support bandage on him. At 3:00 am, the physician decided to transfer Paul to the ED’s bed ward because the nurses needed more time to obtain information about Paul’s home situation in order to enable them to plan his discharge. While he was waiting for the hospital porter to come and transport him to the ED’s bed ward, I sat at his bedside. He held firmly onto my hands. I felt that this gave him a sense of security.

To examine, treat and send Paul home, the physicians and nurses asked questions about his medical situation and about his situation at home. They discovered that he was unable to engage in the conversation in any meaningful way, as he could not produce the answers they needed. Therefore, they also anticipated that he was unable to go home and manage his daily activities without help. The nurses started to search for relatives who might be expected to have knowledge of his situation and who would be able to take responsibility and make decisions on Paul’s behalf. Involving his relatives was therefore a necessity, as information about what had happened, about his home situation, and about his dementia was vital for the medical staff in order for them to fulfil their tasks. The absence of Paul’s relatives was impeding Paul’s clinical pathway and preventing them from giving him effective care.

Paul’ case illustrates how examinations, treatment, and discharge are based not only on the actions of the health professionals but also require foregoing information and contextual, background knowledge based on the active engagement of patients and/or relatives. As Paul was lying in a bed in the treatment room, the physician and nurse practiced their customary routines; they entered the room on and off for short periods of time to perform the necessary examination and treatment routines. As such, it can illustrate how the organizing of the clinical pathway in the ED is based on spending a limited amount of time with each patient in order to facilitate the flow, and is particularly challenging for patients with cognitive disabilities; obviously, such patients might have great difficulties providing answers to concrete questions in a speedy and efficient way. In this case, recognizing the role of the relative and their knowledge of the patient involves not only the patient’s stay in the ED. It highlights how the situation prior to the accident influenced the patient’s clinical pathway during the stay in the ED and the process of planning the discharge.

Paul’s case also illustrates the importance of information and the decisions of relatives: the situation outside the ED had a clear impact on the health professionals’ decisions about Paul’s discharge. In this case, relative involvement would have been desirable and useful, in so far as it would have made the clinical pathway more efficient. In Paul’s case, relatives would have played an essential role in the transfer of responsibility for Paul from leaving the ED to being taken home. In Paul’s case, he had no relatives who could assist him at home, so he ended up being transferred to a municipally rehabilitation center. Cases such as Paul’s illustrate that the relatives’ role can be crucial in order for the clinical pathway and the transfer to the municipal responsibility to be carried out in a timely and secure manner and in accordance with agreed standards.

#### Recognizing the relative on the scene

An important aspect of the practice of involving a patient’s relative is the ability of the participants – patients, relatives, and health professionals – to recognize each other. This was a recurrent theme in our analysis of the cases generally, and it can be illustrated in Debbie’s case.

Debbie, age 22, who suffers from a mild case of autism and lives together with her mother was admitted to the ED on the suspicion of a cervical fracture caused by her horse having pushed her up against a wall. When I met Debbie, she had arrived by ambulance and was lying in a hospital bed in the center of a treatment room. Debbie had been accompanied by her mother Linda, who was sitting at a small table near the wall. I learned from Linda later on that on the day of Debbie’s accident she had just arrived at their home when she saw Debbie walking towards her, crying. Linda noticed visible signs of pressure marks on Debbie’s neck and shoulder. Debbie explained to her mother that while caring for her horse, the horse had pushed her up against a door in the stall. Linda took Debbie to Debbie’s GP who referred her to the ED and requested an ambulance to transport Debbie from the medical practice to the ED. In the ambulance Debbie was put on a spine board and immobilized. Linda felt that Debbie looked as if she felt safe; therefore, Linda chose to go to the ED in her own car while Debbie was in the ambulance. However, because she was worried that Debbie might get agitated in this situation she told the ambulance personnel that Debbie because of her autism might get agitated because she was strapped to the spine board. Linda repeated this information to the nurse on her arrival in the ED because she had noticed that the ambulance personnel forgot to mention it.

From the participant-observation it was evident that whenever the nurse or the physician attended to Debbie, her mother withdrew to the chair by the wall, but she continued to closely observe what was going on. After the physician or nurse had left the room, Linda would walk over to Debbie, or sit and chat with her from her seat near the wall. In the in-depth interview, Linda explained:

I only interfere if it is necessary. […] although I move away from the bed I am still on guard […] I observe both them and Debbie, listen and observe, so I’m working overtime […] There’s no doubt about that. But she is also a grown-up woman of 22 years, so I have to start to withdraw.

In this situation, Linda recognizes herself as a mother of an adult. Debbie is 22 and supposed to be able to participate in the interactions with the nurse and the physician without her support or involvement.

Later on, this understanding of Debbie as a fully competent patient able to interact with the ED staff starts to fall apart. Debbie tells the nurse that she needs to go to the toilet, but she is told that she is not allowed to before the physician is sure that her spine has not been fractured. The nurse then suggested a couple of solutions that Debbie was unable to use. Even though Linda had mentioned to the nurse a couple of times that Debbie was suffering a ‘meltdown’, the nurse apparently did not take this information seriously. Debbie became more and more agitated and as she gets ready to get out of bed anyway, the physician enters the room. In a confrontational manner, he warns Debbie that it is her own responsibility if something happens. Witnessing this, Linda steps into the scene, walks straight over to Debbie and tells the physician angrily, ‘This is not the way you talk to a person who has autism.’ The result of this confrontation was that the physician and Linda stepped outside the treatment room to discuss and make decisions on Debbie’s treatment.

This interference by Debbie’s mother sets the scene for a totally new situation in which Debbie is no longer recognized as able to participate in the interactions with the ED staff on her own behalf. Debbie had been unable to meet the requirements of the practice in which a patient is supposed to comply with the physician and heed the nurse’s instructions. As autism has no immediate external signs, neither the physician nor the nurse recognized that Debbie was suffering from autism, nor that denying her the possibility to use the bathroom was extremely painful and stressful for her. This caused Debbie’s ‘meltdown’. Linda explained that Debbie *‘*gets fiercely angry and then she shuts down and she does something in anger that is not always very clever. One time I saw her standing hitting and kicking a horse because it accidently had stepped on her foot.’

Later on during the in-depth interview with Debbie, she reflected on what had happened in the ED. Asked about what her mother’s help and engagement meant to her in the ED, she replied:

Yes, especially in such a situation [it is important], because if it is an everyday situation. Then I can just, you know, sit down for half a minute, take a deep breath and then try to articulate my way out of it. But in a situation like that [she gets emotional at this point], where you hurt quite a lot and are half-drugged by something they gave you, then there is not much you can do about it. I certainly can’t.

In this situation, Debbie felt a triple sense of misrecognition from the physician and nurse; she was recognized neither as the patient she was; nor as an adult suffering from autism, as someone who needed help to enable her to participate in her own clinical pathway. Finally, Debbie’s mother was not recognized as someone who could assist and contribute to the situation. It was particularly the lack of recognition of her mother that damages her competent participation in her clinical pathway. The Danish healthcare system, as do all such systems, operates with an understanding that adults are capable of managing their own affairs. This understanding undergirds the requirement that the system must respect the patient’s right of self-determination and autonomy. Nevertheless, this assumption can also be a way of harming those patients who need relatives to assist them.

Linda was not recognized by the physician and nurse as a participant, and she was not given a role in this scene where she was not involved in the activities. Linda knew how Debbie might react if she felt stressed, and it was this knowledge that she tried to act upon; she informed the staff about her daughter’s diagnosis, and she assumed that they knew what the diagnosis autism and ‘meltdown’ meant. As the events in the ED unfolded, it became apparent that the nurse did not understand the importance of Linda’s information in relation to her practice of involving Linda in Debbie’s clinical pathway. Debbie’s case indicates that she and her mother had already established their own social practice related to Debbie’s disease prior to Debbie’s admission to the ED and that this practice was extended to the setting in the ED. However, due to norms regarding patients’ rights and autonomy and because there was information that was not communicated or understood, Debbie and her mother were not recognized as participants in Debbie’s clinical pathway.

#### Delayed involvement of the relative

In the following, we use John’s case to illustrate the dilemmas of involving relatives in cases where patients for some reason are reluctant to involve their relatives, thus affecting their transition in and out of the emergency department. John was part of the study for nearly 4 months, and he had been admitted to the ED twice since he agreed to participate in our study.

In his mid-seventies, John appeared to be very social and eloquent when talking about his background. His wife had died of cancer a couple of years earlier. He has a son and a daughter, as well as grandchildren. He also told me that he had celebrated his birthday 2 days earlier. John also mentioned to me that he was on medication for his drinking problem, and that he had stopped taking the medication a few days earlier because he wanted to drink with his friends at his party. The day after the party, however, he felt unwell, and he called the emergency services. An ambulance was dispatched, John was examined in the ambulance, given a sedative, and accompanied back into his house. During the night, John felt worse, and early next morning he called his GP, who requested an ambulance, and he was transported to the ED. During the participant-observation, I witnessed how John began to vomit, his body was shaking, and he was given medication for his withdrawal symptoms.

At one point, John’s daughter, Nina, phoned him, but it was evident from the conversation that he did not tell her that he was at the ED because of his withdrawal symptoms. Instead, he told her that they were examining him because of heart problems. John told me that Nina had been worried because he had not answered the phone the last 2 days. John explained that he did not answer because he felt very ill and did not feel like talking to anyone. Later on, John phoned his son and told him the same story he had told Nina. In the in-depth interview, John explained that he did not want to involve his children, because.

They cannot spend all their spare time and surplus energy on me. They work quite a lot and have children. I do not want them to feel that I am an encumbrance.

After John was sent home, I made an appointment to visit him for the in-depth interview. I rang his doorbell several times and as I was about to leave, John opened the door. He was very confused when he saw me and explained that he was not feeling well. We agreed to make a new appointment for the interview after the summer holidays. In the in-depth interview John gave me permission to interview Nina without him being present, because, as he said: *‘*She should be free to explain the way things are without having to show consideration for me.’

From Nina’s perspective, the period after her father had been discharged after I met him in the ED had been very hard. At some point, John had phoned Nina because he was feeling ill and asked her to come and see him. She called the emergency services for help, and John was again taken to the ED with an ambulance. Nina said that John had only wine and yogurt in his fridge. The ED had promised to contact Nina when they discharged John, but Nina had to phone the ED herself, only to learn that they had already sent John home in a taxi. Later that afternoon, she received a call from the home health aide service, informing her that John was not in his house. The front door was open, the television was on, there was food on the table, but John’s car was gone. Nina suggested that the home health aide return a half hour later, but John was still not at home. Nina’s husband offered to search for John. Failing to find him, he phoned the police. Shortly afterwards, the police called back and told them that John was in an ambulance on his way to the ED.

The next day, a nurse from the ED telephoned Nina because John wanted Nina to take him home. Nina told the nurse that she was worried that John was unable to take care of himself and that the same thing would happen again. The nurse told her there was no reason to keep John in the ED because they had observed him and arranged for the home help service to visit and to have his meals delivered. When Nina collected her father, she received no information from a nurse on his condition, although she thought that John looked as if he was affected by the medication he had been given. Despite this, the nurses in the ED did acknowledge that John was unable to care for himself; they arranged for additional support after the discharge. In the interview, John told me that after his last visit in the ED, they were worried about his state of mind, and that a district nurse visited him several times during the day in order to make sure that he took his medication. Even though John did not want to involve his children in the treatment of his alcoholism, he involved Nina in practical issues such as transporting him from the ED. Nina was not initiated into the details of the discharge plan, and she was not recognized as someone who could contribute to carrying out this plan. It was only when the home help services could not find John that Nina was called to the scene and suddenly given responsibility for locating John. The example shows the various levels of involvement [14]. Here we can see that John ‘curates’ his relative’s involvement by deciding when to involve Nina in practical activities such as transport while excluding Nina from treatment of his drinking problem, assuming that this may endanger their relationship. In my interview with Nina, she explained how she was initially involved when things went wrong. When she later became involved, this created feelings of concern, helplessness, and powerlessness and at the same time made her feel like she was a parent for her own father.

During the interview, Nina explained that she thinks that John’s reluctance to involve his children in his problems is linked to his earlier life, when his wife was the one who had to deal with John’s drinking. John and his wife had no practice of involving the children in John’s alcohol abuse, making it difficult for Nina to confront her father. It was only when her mother became critically ill with cancer that Nina and her brother became aware of John’s alcoholism. Nina knew from her mother that John got angry if he was confronted with this. Nina did not think that it was appropriate for her, as his daughter, to talk to John about his alcohol abuse because she felt that it was an expression of lack of respect for her father and that it could endanger their relationship [12]. The following interview quote illustrates the responsibility Nina feels toward her father:

I have a demanding job and a busy everyday life, so I’m not able to follow up on him as often as I’d like to. And then these situations happen, where things rapidly go downhill because you cannot oversee everything all the time. […] Then at one point, a nurse called me. […] I had given them my phone number and said that I would like to be involved because I want to help my father. [….] There has never been anyone from the hospital who had called me before. Therefore, I first became aware that he had returned home again when we contacted him afterwards. He has also had problems with his mobile phone because it runs out of power. You get anxious if you cannot get hold of him.

Nina and her husband said that the episode where they had to call the police became a turning point for John. It made him realize that he needed help. From the interview with John, it was apparent that he also shared this understanding of the episode. In the course of these events, John had received valuable help from his GP, who had visited him at home a couple of times. The GP had agreed with John to call Nina and invite her to a meeting with him, John, and the district nurse. Nina participated and as a result of the meeting, the GP referred John to a psychiatrist, suspecting that John was suffering from depression. As it turned out, the meeting and the GP’s involvement of Nina initiated a different form of involvement practice that enabled Nina to participate in John’s treatment. She now accompanied John to appointments at his GP, the psychiatrist, and to various examinations.

John’s case shows how issues of patient confidentiality can pose an obstacle to the relatives’ involvement and to relatives’ access to information about the patient’s situation [12, 35]. Even though Nina, in her conversation with the nurse from the ED, expressed concern about John’s discharge based on prior experiences and directly said that she wanted to get involved, this request for involvement was not reciprocated by the nurse. There was no meeting between the ED staff, Nina and John. It was only later in the pathway that John’s GP intervened and facilitated the involvement of Nina. Hence, John’s case highlights the importance of health professionals’ central role in the involvement of relatives, as they are able to facilitate or hinder the involvement of the patient’s relatives at several points in the clinical pathway.

To sum up, the cases of Paul, Debbie, and John illustrate different practices of involving relatives in the patient’s clinical pathway. The patient’s and relative’s involvement (being seen, obtaining information, being contacted, being consulted) is not only an event that takes place when they are being treated for the acute condition in the ED. The results show that there is also a ‘before’ and ‘after’. In Paul’s case, for example, the need for his relatives made their involvement crucial in order for the medical staff to complete his pathway in a safe manner and in accordance with agreed standards. Debbie’s case highlights that she and her mother had an already established potential for involvement prior to Debbie’s admission to the ED, related to her autism and to the fact that Linda was already caring for Debbie. John’s case highlights the importance of the health professionals’ influence on the involvement of relatives; health professionals, especially those who are empathetic and know their patients’ life situation, have the possibility to facilitate or hinder the involvement of the patient’s relatives at several points in the clinical pathway.

#### Involving relatives: before, during, and after

**Fig. 1:**
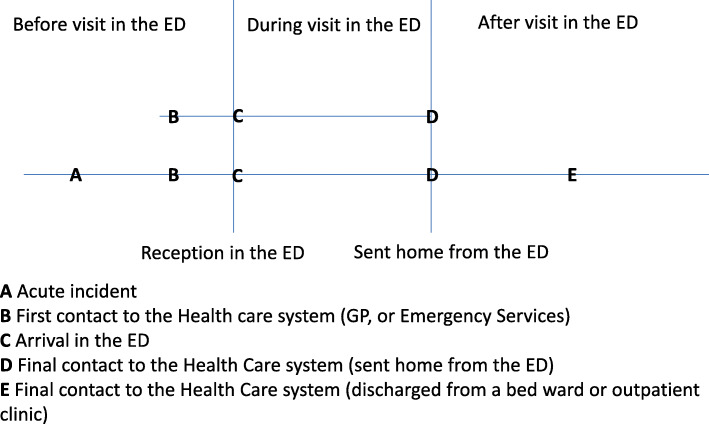
Illustration of the extended acute ambulatory clinical pathway

A key finding of our analysis is that the involvement of relatives does not only pertain to the practices within the ED. In Fig. 1 the upper line (from B to D) illustrates how the acute clinical pathway in the ED is usually thought of. However, this line illustrates only one ‘stop’ on of the complete acute ambulatory clinical pathway [7]. The analysis points toward the need for a more integrated and contextualized understanding of relatives’ involvement, as it takes place along an extended acute ambulatory clinical pathway. This extended pathway is illustrated in the lower line in Fig. 1, from before A (the acute incident) to D or E (when the patient is sent home from the ED or discharged from other departments). We found that the practice of involving relatives in the ED could not be isolated from other practices or factors grounded in situations prior to or after the visit in the ED.

## Discussion

We found that involving relatives in the acute clinical pathway was influenced by practices and relationships within as well as outside the ED. Relatives’ involvement was thus influenced by patients’ and relatives’ on-going relationship and practices, of their experiences from prior visits to the ED or other hospital departments, as well as by the regulated and routinized practices in the ED.

Relatives are often vital partners in the patients’ clinical pathways in the ED, a finding seen in other studies of health care [3, 4, 8, 12, 13, 36]. Our study points to the fact that even when the patients’ relatives were not physically present, they were nevertheless involved at different stages during the clinical pathway (before, during, after the acute incident) for shorter or longer periods, or via telephone, during the patients’ stay in the ED. In accordance with Aarhus et al., we found that it was not always the closest relative who accompanied the patients and remained with them for hours of treatment, tests and waiting [14].

In comparison with studies of relatives’ involvement in other areas of healthcare, such as dementia [13], psychiatric care [12], and cancer [14, 37], our study confirms the complexity of involving relatives in the ED setting. We found that there were great differences in the extent to which the patients wanted to involve their relatives. Even though a patient had close relatives, these relatives were sometimes unable to fulfil the role of a relative in the patient’s clinical pathway because they could be at work, lived far away, were sick themselves, or because the patient did not want them to be present.

In addition to these differences in the relatives’ presence in the ED and the extent of their involvement, our analysis pointed to two central factors affecting involvement practices in the ED: ‘unpredictability’ and ‘knowing the patient’. These factors, we argue affect how the relatives are recognized as participants in the patients’ clinical pathways.

The unpredictability factor differs for ED staff compared to patients and relatives. For the ED staff, unpredictability is related to the inherently unplanned patient inflow and to practices or events occurring outside the ED: the referring practices from the GPs, the occurrence of major accidents, and the ability of ED staff to refer patients to out-patient treatment. All these factors affect the primary ED ‘mission’, which is to keep the flow of patients running smoothly. Our findings indicate that for the ED staff, unpredictability was countered by deploying the routines attached to their professional practices ([13] , p. 10). In Paul’s case, for example, the physician and nurse practiced their customary routines, entering his room on and off for short periods of time to perform examination and treatment. This stress on routine was also found by Häikiö et al., who observed how health professionals performed their activities in patient’s clinical pathways in order to meet ‘the specific needs that fall within the scope of their service’ ([13] , p. 10) and involving relatives did not seem to be within that scope.

Unpredictability for patients’ and relatives’ took on a quite different character. It comprised their sense of temporal and existential uncertainty about when the examinations were actually completed, when they would be sent home, or how their diagnoses would affect their future. This uncertainty was linked to the patients’ and relatives’ (understandable) lack of knowledge about the professional practices of the ED. This kind of uncertainty, we argue, is an effect of the way the acute clinical pathway is constructed. Hence, as Checkland et al. found that patients and relatives were ‘being on, put into, flowing through, led along, moving down pathways implying motion, but also passivity, controlled by the parameters of the pathway to which they have been assigned’ ([38] , p. 416). In this vein, we can argue that the patients’ and relatives’ sense of uncertainty is linked to their having no choice but to ‘follow along’ a clinical pathway set by others. It is a clinical pathway where the patient does not know the endpoint, nor the route, nor how long it will take to get there. Little wonder that some patients become frustrated or experience ‘meltdown’.

In the ED setting, uncertainty about the clinical pathway of their treatment tended to leave the relatives invisible, regardless of whether they were present or not, and regardless of whether they stayed on the ward for few minutes or several hours. The relatives were involved if they were needed, but this need was determined only from the perspective of the ED staff. This contrasts what Aarhus et al., found in their study of fast-track diagnostic cancer pathways where patients were actively encouraged to bring a relative to the consultations for both practical help and emotional support [14]. In their study, this encouragement is acknowledged by the patients. The important aspect, however, is that the cancer treatment staff view the relatives as an essential part of the cancer patients’ clinical pathway. This visible status of the relatives was not mirrored in the ED clinical pathway. On this basis, unpredictability is not only a contextual factor or an inherent aspect of the acute clinical pathway. Rather, unpredictability prevents relatives’ involvement in the clinical pathway. By drawing on an understanding of involvement practices as a situated practice [26], we have shown how involvement operates in context. We found, that involvement requires being recognized as a participant [28] because it is only this recognition which enables the individual to be engaged, to act in the care setting which comprises the acute clinical pathway [26].

Knowing the patient, the other central part of recognizing the patients and relatives as participants, relates to having knowledge about relevant factors during the period before the acute incident occurred, i.e. the situation at home, everyday experiences, and what kind of relations were in place before the acute incident. Häikiö et al. refer to these practices as family practices, meaning, that ‘from the perspectives of family caregivers they are involved in supporting and caring for their relative because they are family or friends who share histories, identity and often homes’ ([13] , p. 10). They point out that the contributions of relatives to the patient’s clinical pathway are often invisible to or ignored by healthcare professionals, as already shown (ibid.). Aarhus et al. describe these processes as ‘the relatedness between patient and relatives’ ([14] , p. 116), and our findings confirm the importance of this relatedness.

We found, this knowledge of the patient was not always sought by the ED staff. As shown in Debbie’s case, involvement of the relatives was considered unimportant until the staff obtained unique knowledge of the patient. The reason why this knowledge was not sought in this case was related to other involvement practices, namely norms regarding adult children’s integrity, patient confidentiality, and the simple fact that some relevant behavioural syndromes are not immediately visible in the ED setting, or are first visible after some tense interactions. In Debbie’s case, the staff acted on assumptions that these norms should remain valid, and consequently, Debbie’s mother was not recognized as a valued participant who could contribute essential information about Debbie’s state. She was overlooked until she spoke up. The effects of mothers’ involvement in their young adults’ pathways when a young person aged 16 is transferred from a children’s ward to a ward for adults was described by Allen et al. [35]. They found that there are clearly ‘issues of confidentiality which prohibit parents having access to the content of private consultations between their child and care providers’ ([35] , p. 999) even if the children want their parents to be involved. From this we can conclude that in the healthcare system, adults are expected to act as a patient who can handle the situation themselves. In Debbie’s case, the staff did not ask Debbie if she wanted her mother involved, nor did Debbie insist. The problem of cross generational involvement was also present in John’s case, which corresponds to the findings of Lund et al., that ‘caregivers [relatives] with relations as adult children of, and siblings to, the patients, younger caregivers (in particular 18 – 49) and caregivers to younger groups of patients experienced most problems regarding a range of interaction aspects’ ([37] , p.1732).

In Paul’s case, on the other hand, knowledge of the patient was actively sought by the staff because completion of his clinical pathway in a safe and timely manner was dependent on whether his relatives could help him when he returned home. This means that to become involved, relatives must be recognized as participants in the patients’ clinical pathways in the ED. Earlier we posed the question of what kind of involvement of relatives is needed in the acute ambulatory clinical pathway in order to fulfil the potential of involvement? On the basis of our analysis, we have found that it is not only important for the ED staff to recognize the perspectives of the relatives but also that they recognize that involvement practices are already in place even before the acute incident. Recognizing and asking about these pre-existing practices is therefore an essential aspect of enabling the relatives to become active participants in the patient’s clinical pathway ‘journey’.

### The practice of involving relatives

In their recent study, Checkland et al. argue that the ‘metaphor “care pathway” translates into a linear, unidirectional pathway that moves from a – b’ ([38] , p. 415). This corresponds well with the institutional definition of the ‘acute ambulatory clinical pathway’ as comprising ‘all the activities, contacts or events that patients go through in connection with the treatment of a certain health condition from the first to the last contact with the health care system’ [7]. ED medical staff have to ensure that patients are diagnosed, treated, and discharged or admitted to another ward as quickly as possible [19] in a flow culture [20] that may be unpredictable and where the time pressures are often intense [18]. The medical staff thus handled the patients’ ‘specific needs that fall within the scope of their services’ [13] in order to expedite them along the pathway. The staff’s practices give an impression that patients’ and relatives’ are moving along a bureaucratic processing pathway, or what has been described as ‘patients, once “on” a pathway will move “seamlessly” through its stages’ ([38] , p.416). In the ED this means discharging or admitting patients to another ward as quickly as possible in order to get the flow running. This way of organizing patient care in a linear fashion, Checkland et al. argue, may not meet the individual patient’s needs, in so far as the patient’s condition requires the availability of different services at different times [38], as our study also confirms.

In an environment like the ED described above, we found that the conditions for the involvement practices of the patient’s relatives are challenging, especially for patients who needed combinations of emotional and practical support that only close relatives could provide. Aarhus et al. concluded that the relatives’ involvement in fast-track cancer pathways amounted to an instrumentalist view of relatives (‘relatives as appendage’) ([14] , p. 116), because the medical staff’s practices ‘implied a short-term view focused on the immediate situation and the possible contributions of relatives to handle it’ (ibid.).

We have argued that in the ED, there exists a ‘clinical pathway’ [7, 38] driven by the professional practices where the relatives’ involvement is instrumentalized [14]. However, there is also a ‘patient pathway’ where the practice of involving relatives is shared by patients and their relatives over time.

There is growing acknowledgement of the importance of involving relatives as partners in patient care, but there is little understanding of the integrated nature of the acute clinical pathway especially as it operates in the more tightly organized, time-pressured ED professional practices. Rather than relatives’ involvement simply being an obligation on behalf of the ED, a right asserted by the patient, or a moral and emotional engagement on behalf of the relatives [5], the practice of involving relatives should be viewed as a situated practice constituted in relation to other practices (both institutional and individual). Our findings, therefore, point to the importance of viewing the patient pathway as an extended pathway that concerns the time before, during, and after the time spent in the ED (see Fig. 1). The clinical pathway, therefore, is just one section of a much longer patient pathway that begins and ends in the patient’s home.

We have shown how individuals become participants through a process in which their specific knowledge, identity, and social membership are acknowledged. It is this acknowledgement, this recognition, that enables people to act and ‘subsequently perpetuate and transform practices through their actions’ [27]. Hence, we should underscore that the practice of involving patients’ relatives is one among many other practices that are performed in the ED, and that these relative involvement practices are themselves part of other practices performed in the ED setting.

### Methodological implications and limitations

Another contribution of our study is methodological. In order to understand the challenges of involving relatives, we sought in-depth knowledge on how the processes of involving relatives unfolded in the acute ambulatory clinical pathway in the ED. To do this, we examined how patients and relatives experienced and dealt with the interactions between patients, relatives, and health professionals in the patient’s acute ambulatory clinical pathway. We conducted an ethnographic case study consisting of various data sources: participant-observation, informal interviews, telephone interviews to follow up on all patients after discharge from the ED, and semi-structured in-depth interviews with participants, both patients and relatives.

As a qualitative study conducted at a single hospital department in the context of the Danish health system, our study has some limitations, among others, limitation such as generalizability of the findings. However, we have attempted to outline the contextual factors in order to make transparent the basis upon which we have drawn the conclusions. The study was designed by the two authors in collaboration, but the fieldwork and interviews were conducted by the first author. The first author, being an occupational therapist and health anthropologist, had a pre-understanding of the organization of the Danish health system as well as her experiences of cooperating with patients with disabilities and their relatives might have influenced the focus of the observations.

Participant-observation proved very useful in obtaining in-depth knowledge of the patients’ and relatives’ experiences, feelings, and perspectives as they unfolded. In Paul’s case, the first author even came to act as a form of a ‘stand-in relative’. By spending time with Paul, and listening to his stories instead of asking questions, bits and pieces of the time and actions that led up to his accident as well as his family background became clear, and this information was made available to the nurses and physicians. Spending time with him also calmed him down and contributed to preventing him from moving his arm or getting out of bed to go home.

### Perspectives and implications for the practice of involving relatives in acute ambulatory clinical pathway

Our findings have raised the question of what kind of involvement of relatives is needed in order for the clinical pathway to move patients along effectively.

This study has contributed with in-depth knowledge of the processes by which relatives are involved (or ignored) in the everyday practices in a Danish ED. The implications for clinical practices are as follows:

- The relatives already have different roles and relations with the patient prior to their arrival at the ED, and some of these roles come into play when the patient is admitted to the ED. Therefore, the experiences of the patients and relatives before their arrival in the ED are important. Knowing the patient, knowledge of existing involvement practices, knowledge of how relatives can enable the patient to participate in their pathway are all essential in ensuring that the patient is treated effectively and in timely fashion.
- The patients and relatives feel that they are the ones who initiate and engage themselves in the practices related to the clinical pathway. Based on the understanding of involvement practices organized by means of many different constituent elements, i.e. procedures, statements, understandings, knowledge, engagement, the body, and objects which work in mutual relation with each other [25], it is important to acknowledge the involvement of relatives as a mutual practice in which all the participants – staff, patients, relatives -- recognize each other. The ED should not be an arena for a ‘struggle for recognition’.
- The ED staff’s reluctance to involve relatives in the clinical pathway in turn prevented the relatives from fully participating during the patients’ stay in the ED, as well as after they had left the ED. Relatives, who are not involved in the ED, may become unable to help the patient to cope with their acute situation during and after the stay in the ED.
- Based on our findings, it might be advisable that the medical staff ask the patients on arrival who has accompanied them in the ED and if and in what way they want their companions involved in the treatment process. The staff should also routinely inform the patients and relatives, who are normally unfamiliar with ED procedures, what the possible next steps might be, thereby aligning mutual expectations regarding treatment, waiting time, etc.

## Conclusions

This study has sought to show that the practices regarding involving relatives in the acute clinical pathway are influenced by practices and relationships before, during, and after the stay in the ED. Involvement of relatives does not start when the patient arrives in the ED. Rather, relative involvement is embedded in ongoing practices already in place before the acute incident occurs. Involvement of relatives is influenced by prior experiences and everyday life as well as by the regulated and routinized practices that begin when the patient arrives at the ED for initial examination, diagnosis, treatment, referral and discharge. On this basis, we have concluded that involving relatives’ entails recognizing the actors (patients, relatives, and medical staff) as full-fledged, equal participants in the common practices acted out in the clinical pathway.

Related to this, we have pointed to differences in the relatives’ presence and ‘status’ as important but not the only explanation as to why involving relatives in the acute clinical pathway is challenging. The analysis points to two central aspects of the involvement practices: namely, the unpredictability of the acute pathway in the ED and the (lack of) time to get to know the patients. These factors form the keys to understanding how involvement of relatives can be performed and made more effective; involving relatives’ entails being recognized as participants in the patients’ clinical pathways. While there is growing acknowledgement of the importance of involving relatives as partners in patient care, we need more understanding and appreciation of the integrated nature of the acute clinical pathway in the ED. Involving relatives will lead to better care and treatment of patients.

## Supplementary Information

**Additional file 1.**

## Data Availability

The dataset, consisting of detailed field notes and interview transcripts in Danish, cannot be completely de-identified, and thus cannot be made available upon request. This is to ensure the confidentiality and anonymity of the Emergency Department and the individual participants. We have discussed this issue with The Danish Data Protection Agency, and according to them, because we are unable to de-identify the dataset sufficiently, we are unable to make them available upon request to the corresponding author.

## Abbreviations

ED: Emergency department
GP: General Practitioner

## Footnotes

1 The ED physicians were specialized in other fields than emergency medicine. Therefore, they are referred to as physicians and not specialists.

2 In Denmark, it is the patient’s GP’s, the doctors from the emergency services, or the emergency operation control center who refer the patient to the ED.
