# Supplementary material for: Involvement un-enabled? An ethnographic study of the challenges and potentials of involving relatives in the acute ambulatory clinical pathway
Source: BMC Health Serv Res. 2020 Nov 26;20:1086. doi: 10.1186/s12913-020-05923-x (PMC7690026; doi:10.1186/s12913-020-05923-x)
Supplement: Supplementary file 1 — Additional file 1. [file 12913_2020_5923_MOESM1_ESM.docx]

###### **Interview guide - Semi structured interviews**

## The interview guides for the telephone- and in-depth interviews consist of open-ended questions designed as a guiding framework to be tailored to each individual interviewee/s (patient, relative or patient and relative) depending on the issues that came up during participant observation and/or telephone interview.

## Short Telephone interview

## How are you?

- How have you managed since we met in the ED a week ago?
- Now, looking back how do you feel about the treatment in the ED? (e.g. the admission, information, waiting time, relative's role, the treatment, the discharge)
- Where there anything especially challenging? (Examples from the ED, from home?) What was helpful? (Examples? Why/How?)
- With reference to their specific situation and relatives present or not – How did your relative assist you? (Examples, feelings, circumstances?)

## Additional in-depth interview in patients' home or researchers' workplace

On a general note: During the course of the interview remember to ask additional questions and follow up on episodes and information from the participant observations and the telephone interviews, explore in detail how the participants experienced and reflected on these episodes, the way they acted, felt.

1. Introduction:

- How have you been doing since we last spoke?
- Short recapture of the project, past common experiences, and introduction to today's interview

2. Reflection on the time before the patient arrived in the ED

- It is now about X weeks since you were admitted to the ED. If you look back, how was it to be admitted to the ED? We have talked about what happened earlier, how do you feel about it now? (Reflections on the acute incident? Feelings of pain, insecurity, uncertainty?)
- Had you ever been to the ED before? Do you have other experiences as a patient? (As a relative, e.g. as a parent, child, sibling? Reflections on role?)
- Do you normally get help from your relatives? If yes, doing what? (Practical, health related?) (Relate this to earlier knowledge from participant observation and the specific situation.)
- What does the relative's help and engagement mean to a patient? Can you see other possibilities for acting/how things could have worked out?

3. Reflections on the time spend in the ED

- Looking back, how do you feel about the treatment in the ED? (and e.g. the admission, information, waiting time, relative's role, the treatment, the discharge)
- How did you feel about your relative being there (or not being there)? What could your relative do to help you? (Ask for specific things they did or assistance they provided: practical, social?)
- To the relative: Did you feel that you could assist the patient (mother/father, sister/brother, daughter/son, friend)? What do you think about being a patients' relative in this situation? Can you imagine another (better) way you, as a relative, could help/get information?
- Can you remember a situation where your relative (or you as a relative) felt able to help, or not able to help? (What happened? How could the staff have helped you to be of assistance?)
- Did you get any questions or directions as a relative (or with regard to your relative)?

4. Turn to what happened after you left the ED

- Can you describe what happened when you left the hospital? (How did they leave, with whom?)
- What happened when you arrived at home (or elsewhere)?
- Did you get any instructions as to what you should do or not? (As patient, as relative? Specific treatment related issues?) How did you feel about it? (Secure, insecure, informed, uninformed?)
- How did you get help after you came home? (Relative? Other family, friends, neighbours, community services?)
- How do you see the future – does the acute incident (or other condition) affect you?

Other things we haven’t mentioned?
